# Alpha-Band EEG Modulation as a Potential Aged-Related Biomarker for Biofeedback-Driven Motor–Cognitive Adaptability

**DOI:** 10.3390/s26144575

**Published:** 2026-07-19

**Authors:** Swapno Aditya, Adam Clarke, Lucy Armitage, Wing-Kai Lam, Winson Chiu-Chun Lee

**Affiliations:** 1 School of Engineering, University of Wollongong, Wollongong, NSW 2522, Australia; ssa439@uowmail.edu.au; 2 Advanced Mechatronics and Biomedical Engineering Research Group (AMBER), University of Wollongong, Wollongong, NSW 2522, Australia; 3 School of Psychology, University of Wollongong, Wollongong, NSW 2522, Australia; aclarke@uow.edu.au; 4 Graduate School of Biomedical Engineering, University of New South Wales, Sydney, NSW 2052, Australia; l.armitage@unsw.edu.au; 5 Department of Sports and Health Sciences, Academy of Wellness and Human Development, Hong Kong Baptist University, Kowloon Tong, Kowloon, Hong Kong, China; 6 Centre for Exercise Science and Medicine (CESAME), Hong Kong Baptist University, Hong Kong, China

**Keywords:** EEG, brain, movement analysis, motor–cognitive, aging, gerontology, IMU

## Abstract

**Background**: Age-related changes in motor–cognitive function are associated with altered neural adaptability, particularly during tasks requiring integration of cognitive and motor processes. Dual-task paradigms are commonly used to assess such interactions; however, gait-based tasks may be unsuitable for older adults with balance impairments. **Objective**: This study used electroencephalography (EEG) to examine neural modulation during a controlled seated foot-tapping paradigm under dual-task and feedback conditions. **Methods**: Thirty-six cognitively healthy participants (18 younger adults aged 18–30 years; 18 older adults aged 65–90 years) participated in this study (mean age: 23.8 ± 2.5 years for young adults, 77.0 ± 4.7 years for older adults). The participants performed three tasks: single-task tapping (ST), dual-task tapping with a flanker task (DT), and dual-task tapping with auditory biofeedback (DT+). Relative EEG spectral power across the alpha, beta, and high beta bands was analyzed across left, right, and midline regions. An aggregate modulation index (AMI) was also computed to quantify overall signal changes across the three tested conditions. **Results**: A significantly greater modulation (*p* = 0.007) was found in younger adults compared to older adults. In particular, younger adults had significant reductions in alpha activity in DT+ relative to DT across all brain regions (*p* < 0.02). In contrast, older adults showed no significant alpha modulation across conditions but exhibited increased high beta activity from ST to DT (*p* < 0.001). **Conclusions**: These findings suggest that alpha-band EEG activity reflects age-related differences in auditory-feedback-driven neural adaptability, supporting the idea that alpha-band modulation may serve as a potential biomarker of motor–cognitive adaptability.

## 1. Introduction

Progressive aging is associated with declines in both motor and cognitive capabilities [1]. These include variability in movements associated with walking [1], diminished coordination [2,3], structural changes in gray and white matter volumes [4,5,6], and reduced performance in attention, sensory perception, and executive functions [7,8]. These age-related changes are closely interrelated. In particular, reductions in cognitive resources and executive control have been found to contribute to poorer motor performance [9]. As a result, dual-task paradigms, in which individuals perform both motor and mental tasks simultaneously, are widely used to probe cognitive–motor interactions and to detect functional declines associated with aging [10].

Reviews of neuroimaging studies have consistently shown higher prefrontal cortex activation in older adults, compared to younger adults, during cognitive–motor dual tasks [11], which has been interpreted as reduced neural efficiency or compensatory recruitment in the aging brain. These age-related differences highlight the potential of dual-task paradigms to reveal changes in executive control and attentional resources associated with aging. However, neuroimaging methods such as functional magnetic resonance imaging (MRI) are constrained by cost, restricted availability, and limited feasibility during repeated motor tasks [12]. Accordingly, there is growing interest in electroencephalography (EEG) approaches that allow neural activity to be monitored during online movement under controlled conditions. EEG provides a non-invasive means of capturing brain electrical activities, such as alpha and beta power and activation patterns during cognitive, motor, as well as cognitive–motor dual-tasking conditions [13]. Changes in alpha and beta power and desynchronization during dual-tasking conditions can practically quantify the neural mechanisms of cognitive–motor interference in aging.

Walking with a concurrent mental task is one commonly used dual-task paradigm to probe cognition–balance interactions and fall risks in older adults [10]. However, such dual tasks are not feasible for people with impaired postural balance, particularly older adults who have a higher risk of falls [14]. Some studies switch to simpler tasks such as seated foot tapping [15]. Dual tasks involving simple motor tasks alone may not be sensitive to age-related changes in cognitive functions [16]. In contrast, comparing neural activity across tasks with varying levels of challenge can reveal how effectively individuals adapt to changing task demands, as reflected in measurable, task-dependent modulation of neural processes [17]. Modulation is important in the area of aging, as it captures how cognitive resources are dynamically allocated across varying task demands [18].

Neural modulation can be further influenced through the use of biofeedback. There are different theories explaining the Biofeedback Effects. Some suggest that concurrent task demands and external feedback can compete for limited neural resources, potentially amplifying the interference effects, especially in older adults who already show constrained neural adaptability [19]. Some other studies suggest that appropriately designed auditory biofeedback devices can entrain the brain, improve motor timing and coordination to reduce variability in rhythmic actions, and enhance sensorimotor integration [20,21]. While seated tapping is a low-risk motor task, it remains unclear whether task-dependent neural modulation can be detected among older adults when such tasks are combined with concurrent cognitive demands and biofeedback. Importantly, it is not currently known whether neural modulation differs between younger and older adults. Addressing this gap is critical for evaluating the potential of such paradigms to reveal aging-related neural biomarkers.

This study employed an EEG sensor approach to assess neural responses during dual-task motor performance with and without auditory biofeedback. By using a safe, seated foot-tapping motor task, the present study aims to measure modulation patterns in older and younger adults. By integrating EEG-derived neural measures with behavioral timing outcomes during a controlled seated foot-tapping task, this study aims to (1) investigate any age-related differences in neural modulation between these two age groups and (2) examine how dual-tasking and dual-tasking-assisted with biofeedback affect EEG -derived brain activities across different brain regions within younger and older adults.

## 2. Method

### 2.1. Participants

Younger adults (n = 18; 18–30 years; mean ± SD: 23.8 ± 2.5 years, 5 males and 13 females) and older adults (n = 18; 65–90 years; 77.0 ± 4.7 years, 8 males and 10 females) were recruited for this study. All 36 participants completed a Standardized Mini Mental State exam [22] and achieved a score of ≥24 out of 30, which showed that they were cognitively normal. They were all right-leg dominant. Inclusion criteria required no self-reported history of neurological conditions (e.g., cognitive impairment, ADHD), musculoskeletal impairments (e.g., Multiple Sclerosis, Parkinsons), or other medical conditions known to affect motor function or EEG signal quality. Younger adults were primarily recruited through university-affiliated channels, whereas older adults were recruited via community outreach associated with university networks and local advertisements. This study was approved by the University of Wollongong Human Research Ethics Committee (Approval No. 112/2023). All participants provided written informed consent prior to participation. Based on the repeated-measures design with estimated effect sizes of 0.4 and a significance level of 0.05, power estimates using G*Power software (version 3.1.1) indicated statistical power values of approximately 0.75 for both age groups. All participants were instructed to refrain from any lower limb exercises for at least 24 h prior to the experiment, and to avoid coffee or other stimulants on the day of the experiment.

### 2.2. Experiment Overview

The experiment employed a repeated-measure design in which each participant completed three task test conditions during a single laboratory session: (1) single-task (ST), (2) dual-task (DT), and (3) dual-task with biofeedback (DT+) conditions. The order of these conditions was randomized across participants to minimize order-related confounds. A 2 min rest interval was provided between conditions. All tasks were performed in a seated configuration. The seated foot-tapping protocol for the young adult cohort has been described previously [21]; the present study extends this framework to include an older adult cohort to examine neural modulation and determine any age-related differences under dual-task and biofeedback conditions.

#### Test Condition Definitions

The three experimental conditions differed in task combination and external pacing:

**Single-task (ST):** foot tapping (self-paced) only.

**Dual-task (DT):** foot tapping (self-paced) + flanker task.

**Dual-task with biofeedback (DT+):** foot tapping (participants asked to synchronize the heard auditory feedback) + flanker task + auditory pacing.

During self-pace foot tapping, participants alternated forefoot and rearfoot contacts with the floor in a structured sequence (two consecutive toe taps followed by two heel taps). The task was performed continuously for two minutes at a self-selected pace. The requirement to switch tapping from heels to their toes introduced additional cognitive load [23]. The flanker cognitive task required participants to identify the orientation of a centrally presented arrow, within 1 s, while ignoring other flanking distractors [24]. Responses were registered via a handheld controller. Familiarization of flanker task was provided prior to the actual data collection. EEG event markers corresponding to stimulus onset and response execution were logged synchronously to support event-related neural analysis. The auditory pacing was provided through a metronome, with frequency individually calibrated before testing based on a one-minute video-recorded foot-tapping trial, in which participants produced repeated taps at a self-selected comfortable rhythm. With the metronome in the DT+ condition, participants were instructed to align their foot taps with the auditory signal as accurately as possible.

### 2.3. Measurements

Continuous EEG was recorded using a 19-channel system (SynAmps2 amplifier, Compumedics Neuroscan, Charlotte, NC, USA) operated with Acquire software (version 4.5.1) during each of the three conditions. Signals were sampled at 1000 Hz and band-limited to DC–70 Hz, with a 50 Hz notch filter applied to attenuate line noise, as previously identified to be effective in minimizing EEG noises [25]. Electrodes were positioned according to the international 10–20 system using tin disk electrodes. Recordings were referenced to the left mastoid (A1), with the right mastoid (A2) recorded concurrently, and a ground electrode placed along the midline between Fpz and Fz. This referencing was performed using linear derivation in Neuroscan 4.5., which generated channels as linear combinations of other channels [26]. Additional electrodes were placed peri-orbitally to capture vertical and horizontal electro-oculographic (EOG) activity, enabling correction of eye-movement and blink artifacts.

The EEG data were processed using Neuroscan 4.5 software. The RAAA algorithm [27], together with the EOG recordings, was used to remove eye blink and movement artifacts. An experienced EEG technician visually inspected that data to identify any segments containing potential excessive muscle activity and remove them. Furthermore, channel interpolation was conducted to correct channels with poor signal quality and excessive noise, whereby affected channels were replaced using the average signal from the two or four nearest neighboring channels. A minimum of 60 s of artifact-free EEG data was selected for analysis. The data was segmented into 2 s epochs and transformed into frequency domain using Fourier transform with a Hamming window. Spectral analysis was then performed to compute relative power across frequency bands. The EEG channels were grouped into left and right hemispheres as well as the midline region. The left hemisphere consisted of 8 electrodes (Fp1, F7, F3, T3, C3, T5, P3, O1), while the right hemisphere included another 8 electrodes (Fp2, F8, F4, T4, C4, T6, P4, O2). The midline region comprised three central electrodes (Fz, Pz and Cz). Such grouping reflected established principles of hemispheric laterization and centralized cortical control [28]. Frontal, central and parietal parts of the brain were not distinguished in the individual hemispheric regions.

Alpha (8–13 Hz), beta (13–30 Hz) and high beta (30–40 Hz) were studied. The alpha frequency band provides information regarding active cortical resources [29] and alpha activity indicates the working or suppression of neural networks during cognitive–motor interference [30]. Beta and high beta frequency bands can reflect cognitive control and inhibition [31] and can provide crucial information regarding cognitive processes and the ability of the brain to maintain cognitive–motor workflow and stability.

Aggregate modulation indexes % (AMIs) were calculated to reflect the magnitude of changes for EEG power across the three experimental conditions. The AMI can help address the dynamic nature of EEG changes across time for better detection of neural patterns than alpha power alone [32]. This was performed by first taking the average EEG relative power across all 19 electrodes and three hemispheres for each tested frequency band and each experimental condition. Then the percentage change between single and dual-task conditions (*Dual-Task Effects*) and dual-task + biofeedback conditions (*Biofeedback Effects*), relative to the dual-task condition, was computed. Finally, the two changes were averaged to calculate the overall AMI value. The calculation procedure is provided in the following:

% change between ST and DT (*Dual-Task Effects*) = (DT − ST)/ST) × 100%

% change between DT and DT+ (Biofeedback Effects) = ((DT+) − DT)/DT) × 100%

Total AMI = (DualTask Effects + Biofeedback Effects)/2

Meanwhile, the reaction time and the number of correct button presses from the flanker task, computed from the Presentation Stimuli Software (Version 1.1.4), were used to calculate the Inverse Efficiency Score (IES). The higher the IES, the lower the overall performance was in the flanker task.

$$IES=\frac{Reaction\;Time\;(ms)}{Accuracy\;(Proportion\;of\;100\%)}$$

The experiment was video-recorded, and the number of foot taps within the two-minute seated tapping was counted. Inter-tap intervals were computed by dividing the two-minute duration by the total number of tap intervals.

### 2.4. Statistical Analysis

All statistical analyses were conducted in SPSS Statistics (IBM v28.0, New York, NY, USA). Between-group differences in neural modulation were assessed using a 2 × 3 mixed ANOVA, with participant group (younger vs. older adults) as the between-subject factor and frequency band (alpha, beta, high beta) as the within-subject factor for EEG. The dependent variable was the aggregate modulation index (AMI), computed to quantify overall changes in EEG activity across conditions. Multiple comparisons were controlled using the Benjamini–Hochberg procedure (false discovery rate, FDR = 0.05).

Within-group repeated-measures MANOVAs were performed on young and older adult groups separately to detect any significant differences in EEG power within each frequency band among the three tested conditions within each of the three brain regions: the left hemisphere, right hemisphere and the midline. If the MANOVA showed statistically significant differences, follow-up repeated-measures ANOVAs were performed across the three task conditions at each brain region for each frequency band. Significant ANOVAs were followed by pairwise comparisons among the three experimental conditions, with *p*-values corrected using the Benjamini–Hochberg procedure. For false discovery rate control using the Benjamini–Hochberg method, *p*-values obtained from pairwise comparisons were first ranked from smallest to largest. The same statistical approach was applied to inter-tap intervals across the three tested conditions. In addition, paired t-tests were conducted to investigate if the differences in IES, reflecting performance in flanker tasks, between the DT and DT+ conditions were significant. Statistical significance was determined by comparing each ordered *p*-value with its corresponding Benjamini–Hochberg threshold (i × Q/n), where i denoted the rank, Q was set to 0.05, and n represented the number of comparisons (n = 3).

In ANOVA/ MANOVA, each dependent variable was considered to be a separate statistical family. FDR correction was applied only to the three tested conditions within each variable. Omnibus testing (e.g., MANOVA followed by ANOVA) was used as an initial screening step, thereby restricting post hoc analyses to a limited set of planned contrasts and supporting the application of false discovery rate (FDR) correction only across the three condition-specific comparisons for each outcome.

## 3. Results

### 3.1. Between-Group EEG Analysis (Younger and Older Adults)

A 2 × 3 mixed ANOVA revealed a significant frequency band × participant interaction for the AMI values (Please see Table 1 and Table 2) (F(2, 68) = 5.76, *p* = 0.007, $n_{p}^{2}$ = 0.26). This effect was further supported by univariate analyses, which also showed a significant interaction (F(2, 68) = 8.17, *p* < 0.001, $n_{p}^{2}$ = 0.194). Pairwise comparison with FDR corrections revealed significant differences in alpha-band AMI values between young and old adults (*p* = 0.03). The large standard deviations in AMI, especially among older adults, indicated high inter-participant variability. The presence of positive and negative responses indicated that while some older adults were able to utilize biofeedback effectively, others experienced inefficient modulation, contributing to the observed dispersion. No significant differences were observed for beta (*p* = 0.28) and high beta (*p* = 0.90) between young and older adults.

No significant main effect of participant group was observed when averaged across frequency bands (F(1, 34) = 3.14, *p* = 0.057, $n_{p}^{2}$ = 0.16), indicating that group differences were frequency-specific rather than global. In contrast, there was a significant main effect of frequency band (F(2, 68) = 5.16, *p* = 0.008, $n_{p}^{2}$ = 0.132), confirming overall differences in modulation across spectral components.

**Figure 1:**
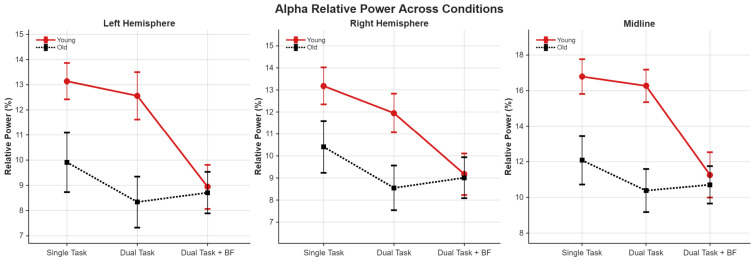
Alpha relative power across different conditions for all hemispheres between young and old adults (mean + SEM).

**Figure 2:**
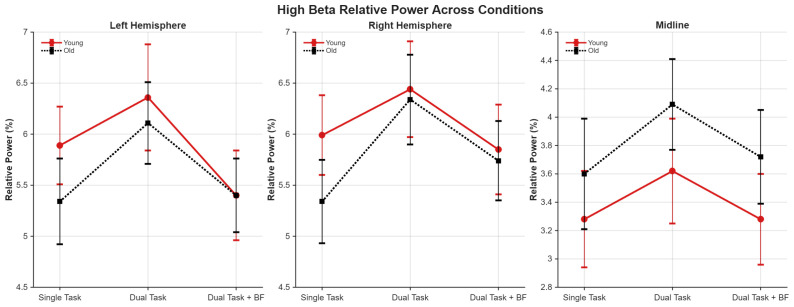
High beta relative power across different conditions for all hemispheres between young and old adults (mean ± SEM).

Relative powers in each of the three brain regions across experimental conditions are shown in Figure 1 and Figure 2. The figures showed that younger adults exhibited higher alpha power than older adults under the ST condition in all three brain regions. This between-group difference remained evident in the DT condition. However, under the DT+ condition, alpha power values were comparable between the two groups.

### 3.2. Within-Group EEG Analysis (Considering Conditions and Brain Region Effects)

**Figure 3:**
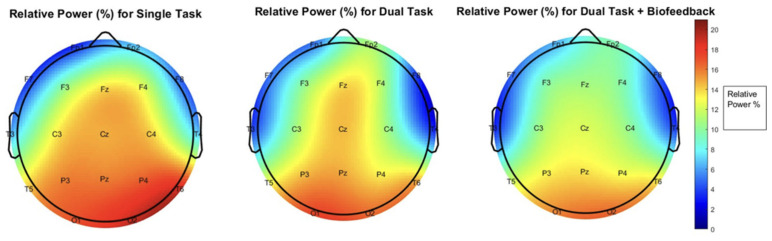
Typical scalp topologies of alpha power over three experimental conditions in a representative young participant.

Among young adults (Figure 3), MANOVA showed an overall significant difference in the alpha frequency band across the three task conditions and three brain regions (F(2, 15) = 4.43, *p* < 0.01, $n_{p}^{2}$ = 0.294). Univariate ANOVA also revealed that there were significant differences across tested conditions in the left hemisphere (F(2, 34) = 9.97, *p* < 0.01, $n_{p}^{2}$ = 0.37), right hemisphere (F(2, 34) = 8.8, *p* < 0.01, $n_{p}^{2}$ = 0.342) and midline (F(2, 34) = 11.02, *p* < 0.01, $n_{p}^{2}$ = 0.4) with the midline showcasing the strongest effect of ($n_{p}^{2}$ = 0.4). Pairwise comparison with FDR correction further showed that participants in the DT+ condition showed significantly decreased relative alpha power in the left hemisphere (F(1, 17) = 3.14, *p* < 0.01), midline (F(1, 17) = 3.01, *p* < 0.01), and right hemisphere (F(1, 17) = 3.68, *p* < 0.02), compared to the dual-task condition. For relative beta and high beta powers, no main significant differences were observed (F(1, 17) = 1.5, *p* = 0.25 and F = 2.9, *p* = 0.052).

Among older adults, MANOVA revealed a main significant effect in relative beta power (F(2, 15) = 3.65, *p* = 0.027. $n_{p}^{2}$ = 0.64), but no significant differences were observed in further univariate tests. MANOVA also revealed a main significant effect in relative high beta power (F(2, 15) = 3.6, *p* = 0.027, $n_{p}^{2}$ = 0.645). Further univariate ANOVA revealed significant effects in the right hemisphere across task conditions (F(2, 34) = 3.64, *p* = 0.047, $n_{p}^{2}$ = 0.177), with higher relative power in the dual-task condition compared to the single-task condition (*p* < 0.01) and no significant differences were observed between dual-task and DT+ conditions (*p* > 0.05). ANOVA did not reveal any significant differences across the task conditions for relative alpha powers (*p* > 0.05).

### 3.3. Flanker Task and Seated Tapping Performance

Younger adults had a lower mean IES (better flanker task performance) in the DT+ condition (965 ± 551, *p* = 0.059), compared to the DT condition (1027.5 ± 566), although the difference did not reach statistical significance (*p* > 0.05). Similarly, older adults had an average IES lower in the DT+ condition (1223 ± 430), compared to the DT condition (1266.85 ± 478).

Meanwhile, younger adults had significantly higher inter-tap intervals (slower tapping) in the DT condition (0.011± 0.0011 s), compared to the ST (0.009 ± 0.0008) and DT+ conditions (0.010 ± 0.0008, *p* < 0.0001). The same trend was observed in older adults, who had significantly higher inter-tap intervals in the DT condition (0.013 ± 0.0018), compared to the ST (0.010 ± 0.0014) and DT+ conditions (0.012 ± 0.0014).

## 4. Discussion

### 4.1. Key Findings and the Seated Tapping Paradigm

This study examined EEG signal modulation across single-task (ST), dual-task (DT), and dual-task with biofeedback (DT+) conditions in younger and older adults. Three key findings emerged. First, the alpha-band aggregate modulation index (AMI) differed significantly between groups, with greater modulation in younger adults, while no group differences were observed in beta and high beta bands. Second, younger adults showed alpha-band suppression in the DT+ condition, relative to the DT condition. Third, older adults showed minimal alpha modulation, but increased beta and high beta from ST to DT, with no further modulation in DT+.

The use of a seated tapping paradigm enabled controlled acquisition of EEG signals by minimizing postural and biomechanical confounds within whole-body movement. This configuration provides a stable platform for isolating sensor-derived neural responses to cognitive–motor demands, allowing EEG to function as a reliable, noise-free outcome measure for quantifying attention, motor control, and feedback processing [33,34]. As such, it would maximize the validity of our human sensing model for studying neural adaptability.

### 4.2. Age- and Condition-Dependent Neural Modulation

Our younger participants demonstrated higher alpha relative power consistently across regions than older participants, which is in line with previous studies [35,36]. Alpha suppression is associated with active attentional engagement and information processing [35,36]. The reduced modulation observed in older adults suggests diminished capacity to dynamically adjust neural responses to increasing task demands. This suggests that alpha-band EEG features provide a potential signal biomarker of adaptability, particularly in distinguishing age-related differences. At the ST condition, the higher alpha power observed in younger adults compared to older adults is consistent with the established age-related reduction in alpha activity [37]. Notably, this study further demonstrated significant alpha suppression in younger adults under the DT+ condition, resulting in comparable alpha power between the two groups. Meanwhile, the absence of group differences in beta and high beta suggests these signals are less sensitive to adaptive changes at the system level.

Examining individual group results showed that younger adults had significant reductions in alpha power in DT+ compared to DT, with consistent effects across the left hemisphere, right hemisphere, and midline, and the strongest modulation observed in the midline region. Consistent with evidence that alpha suppression is associated with active attentional engagement and information processing [35,36], the reduction in alpha power in DT+ reflects a release of inhibition [37] which then allows greater integration of externally provided feedback signals into motor control. Our results suggest that alpha modulation in younger adults reflects the integration of external feedback with higher motor–cognitive adaptability rather than general cognitive load. The introduction of biofeedback likely increased demands for continuous monitoring and error detection [38], leading to enhanced neural engagement reflected in reduced alpha power. Meanwhile, the midline dominance suggests involvement of central control regions associated with sensorimotor integration and executive processing, while bilateral modulation (left and right hemispheres) indicates coordinated engagement of task-relevant neural systems [39].

In contrast, the older adults showed no significant alpha modulation across conditions, suggesting reduced sensitivity of attentional control systems to both task demand and feedback. This is consistent with prior work showing age-related reductions in inhibitory control and alpha dynamics [40]. According to the ‘Compensation-Related Utilization of Neural Circuits Hypothesis (CRUNCH)’ framework [41], older adults may operate closer to their neural capacity limits, limiting their ability to further modulate activity under increasing demands. This may explain the absence of further adaptation in DT+. Instead, older adults exhibited increases in beta and high beta activity from ST to DT, particularly in the right hemisphere. This is partly consistent with previous studies which reported higher right-hemisphere theta and alpha power in older adults during working-memory tasks [42] and the role of the right hemisphere for maintaining resilience to aging [43]. Furthermore, the hemispheric reduction model in older adults as described by Cabzea et al. [44] showed that aging may reduce unilateral neural efficiency, which might promote one certain hemispheric side to work harder or be under more scrutiny. Tasks such as sequential motor function (similar to continuous foot tapping in our experiment) apply loading on the left hemisphere and this may enable older adults to recruit their right hemisphere for functional stability whilst they are doing a cognitive–motor dual task [45,46]. Furthermore, interhemispheric inhibition may activate right-hemisphere activity in older adults when they are under the cognitive load of a dual task [47], which was also what we observed in our findings. Beta activity has been associated with motor control and compensatory recruitment under cognitive load [48,49]. This suggests that older adults rely more on motor-related processes to maintain performance, rather than engaging flexible attentional modulation. However, no further changes were observed in DT+, indicating limited responsiveness to feedback. While auditory feedback has been shown to reduce dual-task cost (Ghai et al. [50]; Park & Lee [51]), the lack of significant modulation here suggests that older adults may have reduced ability to translate external cues into adaptive neural responses.

Overall, the results showed that, while significant differences in EEG alpha modulation were observed between the age groups, both groups exhibited similar patterns of behavioral changes in foot-tapping and flanker task performance, as measured by inter-tap intervals and IES, respectively, across the three test conditions.

### 4.3. Implications and Biomarker Potential

The observed differences between younger and older adults suggest that alpha modulation, as indicated by the aggregate modulation index (AMI), may serve as a plausible biomarker of age-related changes in adaptability. Reduced AMI values in older adults reflect blunted alpha responsiveness, suggesting a diminished capacity to integrate feedback and update motor commands, which is a critical component of functional performance in real-world contexts. This interpretation is consistent with the CRUNCH framework, where aging is associated with reduced neural flexibility and limited capacity to recruit additional resources under increasing demand [41].

Unlike traditional interpretations that link alpha activity primarily to task difficulty, the results demonstrate that AMI-derived alpha modulation is specifically sensitive to the integration of external feedback across experimental conditions, rather than to generic load effects. The graded changes in alpha modulation across task conditions reflects the system’s ability to shift between internally driven control and externally guided adjustment. This suggests that alpha modulation may reflect a functional index of neural adaptability, capturing how efficiently the brain incorporates incoming sensory information to update motor behavior.

These findings have several practical implications for sensor-based applications. AMI is an exploratory metric that has not been formally validated. Its reproducibility, sensitivity, and generalizability across different populations and task paradigms remain to be established. Future studies should therefore evaluate the reliability of AMI. If validated in future studies, alpha-band AMI may be used to: (1) detect early declines in motor–cognitive integration, (2) monitor changes in neural responsiveness over time or following interventions, and (3) support assessment and design of adaptive biofeedback systems, where older adults respond very differently from younger adults.

### 4.4. Limitations

Several limitations should be considered when interpreting these findings. First, the use of a seated tapping paradigm, while advantageous for controlled signal acquisition with reduced movement artifacts in EEG signals, limits generalizability to more complex and ecologically valid motor tasks such as gait or postural control. Although the simplified setup enables clearer interpretation of EEG-derived signals, future studies should extend this framework to dynamic, real-world movements to evaluate how alpha-based biomarkers behave under more functional conditions. Secondly, although preprocessing steps, including artifact corrections, were applied to minimize non-neural contributions, residual electromyographic contamination cannot be entirely excluded. Meanwhile, while the observed EEG changes provide insight into neural modulation, they may also reflect a combination of cognitive, motor, and physiological processes rather than a single underlying mechanism. Furthermore, the present study employed a cross-sectional design, which does not capture longitudinal changes in neural adaptability across individuals. As motor–cognitive decline is a progressive process, future work should investigate how alpha modulation evolves across time within individuals, particularly to validate its utility as a longitudinal biomarker of aging-related change. Typically, biofeedback is defined as the provision of real-time information regarding movement performance. However, there are important considerations when interpreting metronome-based cues within this framework. While metronome signals may be viewed as a simple form of biofeedback [52], some studies suggested that such feedback may contribute to entrainment effects [53], potentially influencing task performance independently of feedback-based error correction mechanisms.

## 5. Conclusions

EEG modulation across single-task, dual-task, and dual-task with biofeedback conditions demonstrates that alpha-band activity is not significantly altered by dual-task demand alone, but is selectively modulated in response to feedback-based conditions, particularly in younger adults. In contrast, older adults showed limited alpha modulation and reduced responsiveness to biofeedback, alongside increased reliance on beta-related activity under dual-task conditions. These results suggest that motor–cognitive adaptability is primarily reflected in the ability to integrate external feedback rather than in response to cognitive load alone. The distinct modulation patterns observed between age groups indicate that aging is associated with reduced flexibility in neural response to changing task demands, particularly in feedback-driven contexts. From an applied perspective, these findings suggest that alpha-band modulation may serve as a potential candidate biomarker of motor–cognitive adaptability; however, further studies are required to establish its validity, reliability, and clinical applicability.

## Figures and Tables

**Table 1:** Relative power (%) and AMI values (%) across 3 experimental conditions (power averaged across all EEG electrodes) for young adults.

Parameter	Single Task	Dual Task	Dual Task + Biofeedback	AMI
Alpha	14.37 ± 3.38	13.59 ± 3.64	9.79 ± 4.25	−14.8 ± 20.40
Beta	16.58 ± 4.24	16.68 ± 4.34	15.67 ± 3.60	−1.9 ± 12.61
High Beta	5.05 ± 1.40	5.47 ± 1.74	4.87 ± 1.56	−6.4 ± 16.83

**Table 2:** Relative power (%) and AMI values (%) across 3 experimental conditions (power averaged across all EEG electrodes) for older adults.

Parameter	Single Task	Dual Task	Dual Task + Biofeedback	AMI
Alpha	10.8 ± 5.17	9.09 ± 4.48	9.47 ± 3.91	2.57 ± 29.80
Beta	15.97 ± 4.70	16.56 ± 4.00	16.19 ± 3.75	−2.1 ± 14.73
High Beta	4.75 ± 1.64	5.51 ± 1.52	4.9 ± 1.44	−10.29 ± 17.71

## Data Availability

The original contributions presented in this study are included in the article. Further inquiries can be directed to the corresponding author.
